# 

**Published:** 1884-04

**Authors:** 


ORIGINAL COMMUNICATIONS:

Address to Graduates of the Philadelphia Dental College. S. H. Guilford .... 169
Iodoform in Dental Surgery. C. F. W. Bodecker .... 177
On the Disposition of Time and its Relation to Fees. A. W. Harlan... 186
Two Cases in Practice. J. L. Williams .... 191
Experiments and Logic. Charles Mayr .... 193

REPORTS OF SOCIETY MEETINGS:

Mississippi Valley Dental Association .... 196
Odontological Society of New York .... 206

CORRESPONDENCE:

From F. W. Dolbeare .... 207
From J. Smith Dodge .... 209
From J. Edw. Line .... 209

EDITORIAL:

Dental Education Again .... 209
Commendatory .... 214
Suggestive .... 214
Nausea .... 215
Archives of Dentistry .... 215
Toothache .... 216
Crowded Out .... 216

CURRENT NEWS AND OPINION:

Another National Dental Society .... 217
College Commencements .... 218
Legislation .... 220
A Simple Operation for Facial Neuralgia .... 221
Registering Vocal Sounds .... 221
An Error .... 221
"It Pays Well." .... 222
Central Dental Association of Northern New Jersey .... 222
Doctor Lord's "Sickles." .... 222
Dental Society Meetings for April .... 223
Specific Disease in the Lower Animals .... 223
Iodoform in Surgery .... 223
Journalistic Births and Deaths .... 223
Removal .... 223

